# Enhanced presynaptic mitochondrial energy production is required for memory formation

**DOI:** 10.1038/s41598-023-40877-0

**Published:** 2023-09-02

**Authors:** Erica L. Underwood, John B. Redell, Kimberly N. Hood, Mark E. Maynard, Michael Hylin, M. Neal Waxham, Jing Zhao, Anthony N. Moore, Pramod K. Dash

**Affiliations:** 1 Department of Neurobiology and Anatomy, The University of Texas McGovern Medical School, P.O. Box 20708, Houston, TX 77225 USA; 2 Present Address: Department of Electrical and Computer Engineering, University of Houston, Houston, TX USA; 3 Present Address: Department of Psychology, Southern Illinois University, Carbondale, IL USA

**Keywords:** Mitochondria, Energy metabolism, Learning and memory, Fear conditioning

## Abstract

Some of the prominent features of long-term memory formation include protein synthesis, gene expression, enhanced neurotransmitter release, increased excitability, and formation of new synapses. As these processes are critically dependent on mitochondrial function, we hypothesized that increased mitochondrial respiration and dynamics would play a prominent role in memory formation. To address this possibility, we measured mitochondrial oxygen consumption (OCR) in hippocampal tissue punches from trained and untrained animals. Our results show that context fear training significantly increased basal, ATP synthesis-linked, and maximal OCR in the Shaffer collateral-CA1 synaptic region, but not in the CA1 cell body layer. These changes were recapitulated in synaptosomes isolated from the hippocampi of fear-trained animals. As dynamin-related protein 1 (Drp1) plays an important role in mitochondrial fission, we examined its role in the increased mitochondrial respiration observed after fear training. Drp1 inhibitors decreased the training-associated enhancement of OCR and impaired contextual fear memory, but did not alter the number of synaptosomes containing mitochondria. Taken together, our results show context fear training increases presynaptic mitochondria respiration, and that Drp-1 mediated enhanced energy production in CA1 pre-synaptic terminals is necessary for context fear memory that does not result from an increase in the number of synaptosomes containing mitochondria or an increase in mitochondrial mass within the synaptic layer.

## Introduction

Mitochondria exist in a complex network that can be rapidly altered to adapt to changing cellular conditions, including mobilizing mitochondria to different cellular compartments and engaging the dynamic processes of fission and fusion in order to meet the constantly fluctuating energy needs of a cell^1^. In the brain, mitochondria play a fundamental role in neuronal survival, homeostasis, and function^2^. It has been estimated that activity-driven vesicle release has one of the highest demands on ATP, requiring 1.64 × 10^5^ ATP molecules per vesicle released, and requiring a neuron to generate 7.1 × 10^8^ ATP molecules for each action potential^3^. Due to this high metabolic demand, mitochondria are often selectively retained in synapses^4–7^. Consistent with high metabolic demands, it has been reported that vesicle release is highest in presynaptic boutons that contain a mitochondria^8^. It is therefore not surprising that several neurodegenerative diseases have been linked to altered mitochondrial function, and that mutations in mitochondrial DNA and mitochondrial protein-encoding nuclear genes are associated with age-related cognitive decline^9–12^.

A large body of evidence indicates that long-term memory formation requires protein synthesis, gene expression, enhanced neurotransmitter release, increased excitability, and formation of new synapses^13–15^, all of which have been linked to mitochondrial function^8,16–20^. For example, it has been found that there are thousands of mRNAs that are differentially translated between synapses and neuronal cell bodies, and that their translation in response to plasticity-inducing stimulation is dependent on energy provided from local mitochondria^16,17^. Furthermore, glutamate can alter mitochondrial morphology and dendritic changes in cultured neurons, and that manipulation of mitochondrial dynamics alters synapse formation^19,21^. In addition to generating ATP, mitochondria also play a key role in calcium buffering^22–26^, and presynaptic mitochondria can facilitate recovery from moderate-to-high synaptic activity by sequestering calcium^25^. Specifically, it has been demonstrated that synapses can switch from glycolytic to oxidative metabolism during periods of high demand, a process dependent on the mitochondrial calcium transporter MICU3 (mitochondrial calcium uptake family member 3)^26^.

Both chemical long-term potentiation (cLTP) of neurons in culture and high-frequency stimulation of Schaffer collateral-CA1 synapses in hippocampal slices have been shown to rapidly increase mitochondrial fission in dendrites^27^. Interestingly, disrupting mitochondrial fission using a dominant negative dynamin-related protein 1 (Drp1) dramatically reduced LTP^27^. While ATP levels in the cell body are unaffected by loss of Drp1, ATP levels are reduced in axons in response to activity, resulting in decreased vesicle recycling^23^. In Drosophila, Drp1 mutations result in synapses lacking mitochondria that cannot maintain normal activity during intense stimulation^28^. Likewise, in Drp1-knockout mice, reduced numbers of presynaptic mitochondria were also observed, and following intense stimulation synaptic transmission was impaired^29^. While these and other related studies have demonstrated a role for Drp1-mediated fission in mitochondrial localization to presynaptic terminals and how their absence can impact neurotransmission, whether mitochondrial energy production is altered as a result of learning in vivo, and if Drp1 plays a role in this process, is unknown.

Recently, an assay has been developed and optimized that allows for the measurement of mitochondrial respiration in discreet brain regions and subregions using small brain tissue punches^30^. Using this assay, we examined if training in a context fear task altered respiration in brain tissue punches, including in the CA1 pyramidal cell layer and the Schaeffer collateral-CA1 synaptic layer, as these regions have been shown to be critical for hippocampal memory formation^31,32^. To specifically examine presynaptic mitochondria function, synaptosomes were isolated and used for respiration measurements. Finally, we used Drp1 inhibitors to assess if the context fear training-related increase in presynaptic mitochondrial respiration was reliant on Drp1, and if blocking Drp1 affected long-term hippocampus-dependent memory.

## Results

### Learning selectively increases mitochondrial respiration in the Schaffer collateral-CA1 synaptic layer

**Figure 1:**
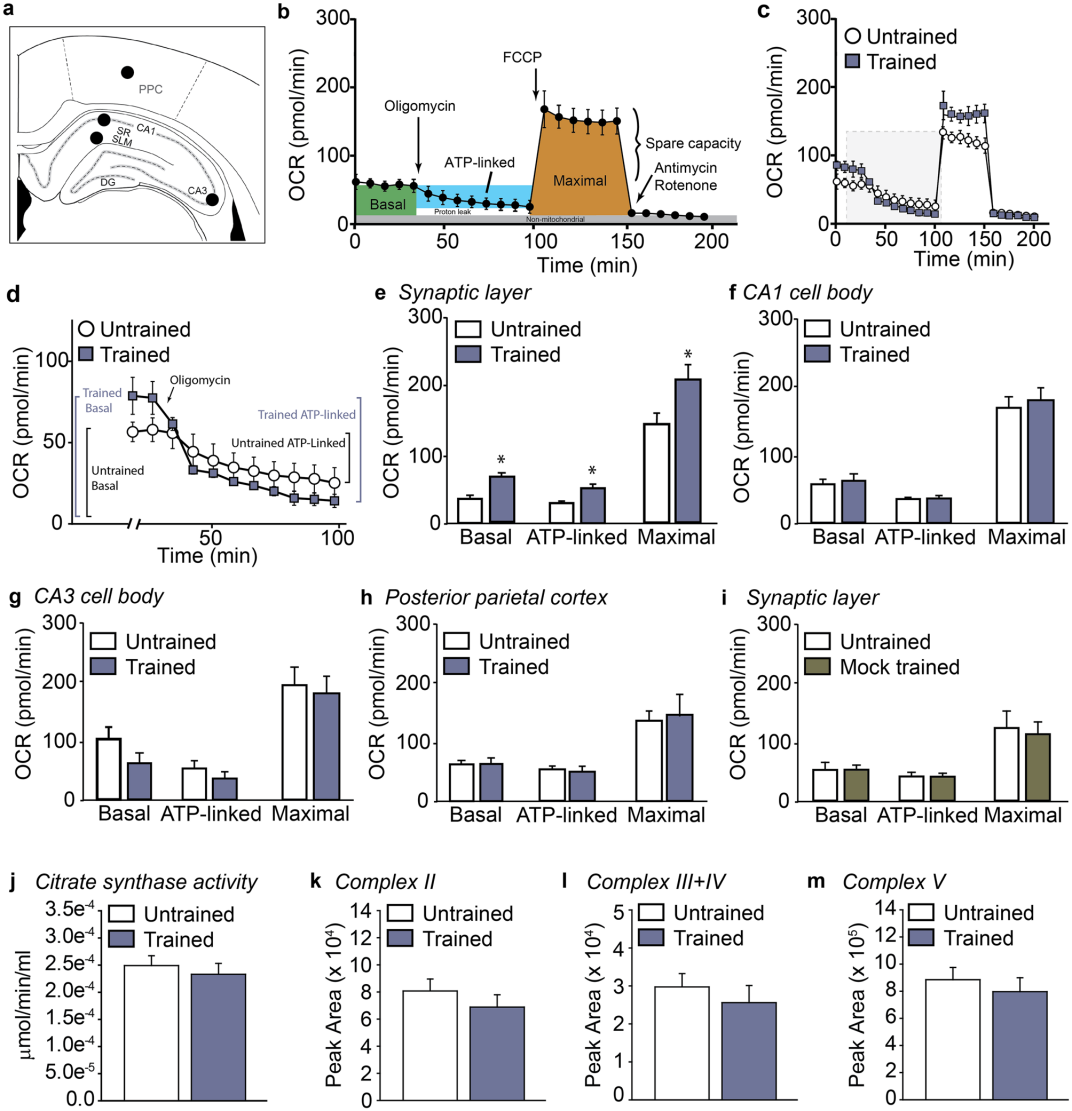
Learning increases mitochondrial respiration in the Schaffer collateral-CA1 synaptic layer. (**a**) One hour after contextual fear training, tissue biopsies from the stratum radiatum/stratum lacunosum-moleculare (SR/SLM), CA1, CA3 and posterior parietal cortex (PPC) were prepared for respiration measurements. (**b**) OCR curve showing the effects of the mitochondrial inhibitors/uncouplers, and respiration measures were obtained. (**c**) Representative OCR curves generated from tissue biopsies of the SR/SLM from rats trained in the contextual fear task compared to untrained controls. (**d**) Expanded view of gray area shown in (**c**) emphasizing training related changes in basal and ATP-linked respiration. Summary data showing basal, ATP-linked and maximal respiration in the (**e**) SR/SLM, (**f**) CA1, (**g**) CA3, and (**h**) posterior parietal cortex in untrained and fear-trained animals. (**i**) Summary data showing that mock training (handling with immediate shock in a darkened fear chamber) does not significantly alter basal, ATP-linked or maximal respiration in the SR/SLM. (**j**) Summary data showing citrate synthase enzymatic activity detected in pooled SR/SLM punches from untrained versus fear-trained animals is not significantly different. SR/SLM tissue punch lysates were also probed using a cocktail of antibodies to detect mitochondrial respiration core proteins for (**k)** complex II (SDHB), (**l**) complexes III and IV (UQCRC2 and MTCO1), and (**m)** complex V (ATP5a). No difference in expression level was detected between SR/SLM tissue punches from untrained and fear-trained animals. Data are presented as mean ± SEM. *p < 0.05.

To assess if learning increases mitochondrial respiration, a group of animals (n = 8) were trained in the context fear task as described in the “Methods” section. One hour following training, animals were euthanized and biopsy punches prepared from the stratum radiatum/stratum lacunosum-moleculare (SR/SLM), CA1, and CA3 pyramidal layers, were used for respiration measurements (Fig. 1a). Tissue punches were also excised from the posterior parietal cortex to serve as internal controls, as this region is not critical for either context or cue-elicited fear^33^. The one hour delay period was selected to allow time for acute, learning-related events that are ATP-dependent, such as increased Erk1, PKC, and CaMKII phosphorylation, to occur^34^. This delay period is also consistent with the recent demonstration that chemically induced LTP caused a rapid increase in CaMKII- and Drp1-dependent dendritic mitochondrial fission events, which were maintained for at least 1 h^27^. Naive animals (n = 8) were used for baseline controls, as opposed to animals placed in a non-shock cage, in order to minimize possible interference due to learning that may occur when animals are placed into a novel context. Figure 1b shows a stylized representation of the effects of the mitochondrial inhibitors/uncouplers used in the tissue respiration assay on different aspects of oxidative respiration. The representative OCR curves in Fig. 1c show that different aspects of mitochondrial respiration in the synaptic layer of fear-trained animals were altered compared to untrained animals. Figure 1d shows an enlarged view of the training curve demonstrating how basal and ATP-linked respiration were calculated. Summary results presented in Fig. 1e showed that context fear training significantly increased basal (pre-oligomycin) (*t* = 2.177, *p* = 0.034), ATP-linked (post-oligomycin) (*t* = 3.319, *p* = 0.002), and maximal (post-FCCP) (*t* = 2.517, *p* = 0.016) respiration in the SR/SLM, which is the location of synaptic connections between the Schaffer collateral axons and CA1 apical dendrites. When the percent of maximal respiration that is in reserve {spare capacity (post FCCP—basal) OCR/maximal OCR (post FCCP)} X 100 was calculated, we observed a decrease after training (naïve: 75.42 ± 0.05%; trained: 62.58 ± 0.03%; *t* = *2.327, p* = 0.023), suggesting that spare capacity was being used to increase basal respiration after training. In tissue punches taken from the CA1 (Fig. 1f) and CA3 (Fig. 1g) pyramidal cell layers, training did not alter any of the mitochondrial respiration metrics examined. Posterior parietal cortex OCR was not significantly different between trained and untrained rats (Fig. 1h).

Although we only observed increased mitochondrial respiration in tissue punches taken from the SR/SLM, it is plausible that factors other than learning contributed to these changes, such as differences in handling or stress caused by the fear task. To address this possibility and minimize the possibility of contextual learning, we generated a group of mock-trained animals that received the same handling and foot-shock as the fear-trained animals, but were not allowed time to explore the training chamber. Figure 1i shows that when SR/SLM tissue punches from the mock-trained animals were compared to naïve controls, no differences in either basal, ATP-linked, or maximum OCR were observed. Consequently, naïve rats were used as baseline controls for all subsequent experiments. To assess if fear training may have changed mitochondrial mass in the SR/SLM, we assessed citrate synthase enzyme activity as a surrogate measure for total mitochondria^35^. Citrate synthase activity was directly proportional to tissue input (Supplemental Fig. S1a,b). Figure 1j shows that citrate synthase activity in total cellular homogenates generated from SR/SLM tissue punches taken from fear-trained animals was not significantly different to that from untrained animals (*t* = 0.245, *p* = 0.812). We further assessed the expression levels of mitochondrial core respiration complex proteins in the SR/SLM homogenates. Similar to citrate synthase activity, we found no difference in the immunoreactivity of Succinate Dehydrogenase (SDHB; Fig. 1k, complex II; *t* = 0.941, *p* = 0.374), Ubiquinol-Cytochrome C Reductase Core Protein 2 (UQCRC2) and Mitochondrially-encoded Cytochrome C Oxidase 1 (MTCO1) (Fig. 1l, complex III and IV; *t* = 0.723, *p* = 0.491), or ATP synthase F1 subunit alpha (ATP5F1a; Fig. 1m, complex V; *t* = 0.643, *p* = 0.538) in SR/SLM lysates from untrained versus fear-trained animals. Finally, we did not detect any statistical differences between untrained and fear-trained animals when tissue sections were immunostained for the mitochondrial marker translocase of outer mitochondrial membrane 20 (TOMM20; Supplemental Fig. S4).

### Hippocampal synaptosome respiration is increased following training

**Figure 2:**
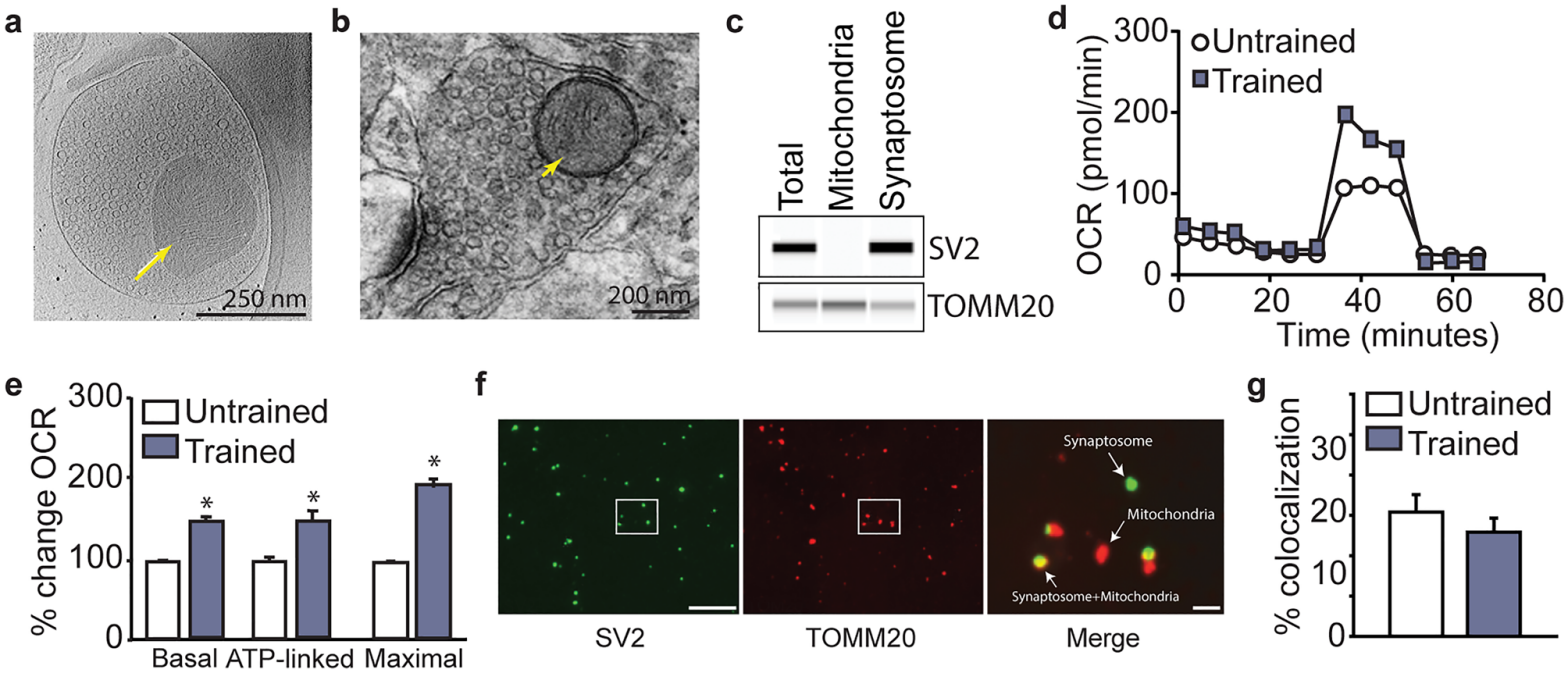
Oxygen consumption rate of hippocampal synaptosomes is increased following training. (**a**) Cryo-EM and (**b**) TEM images of an isolated synaptosome and a presynaptic terminal, respectively demonstrating the presence of synaptic vesicles and mitochondria within these structures. (**c**) Representative capillary western showing the presence of the synaptic vesicle protein SV2 and the mitochondrial protein TOMM20 in synaptosome extracts, whereas only TOMM20 is detected in isolated mitochondria (full-length capillary images are shown in Supplemental Fig. S3). (**d**) Representative OCR curves generated from synaptosomes isolated from the hippocampi of rats trained in the contextual fear task versus naïve rats. (**e**) Summary data showing that fear training significantly increased basal, ATP-linked, and maximal respiration in synaptosomes. (**f**) Confocal images showing SV2 (left panel, green) and TOMM20 (center panel, red) immunostaining of an isolated synaptosome preparation. The boxed area is shown at higher magnification to illustrate SV2 + TOMM20 signal colocalization (right panel, yellow). (**g**) Summary data showing the percentage of synaptosomes containing mitochondria in preparations isolated from naive and contextual fear-trained rats. Data are presented as mean ± SEM. *p < 0.05. Scale bars represent (**a**) 250 nm, (**b**) 200 nm**,** (**f**) 20 µm (left panel), and 2 µm (right panel), respectively.

Although OCR measurements using tissue punches maintains mitochondria within their native intracellular environment, it does not reveal if the increased respiration observed after fear training in punches taken from the synaptic layer is occurring at neuronal synapses. To examine this, we isolated hippocampal synaptosomes from trained and naive animals. A representative CryoEM image of a synaptosome isolated from a rat hippocampus is shown in Fig. 2a. Similar to that seen in a transmission electron microscope (TEM) image of a presynaptic terminal within the SR/SLM (Fig. 2b), the isolated synaptosome contains numerous synaptic vesicles and a mitochondrion. Western analysis of isolated mitochondria and synaptosome preparations show that while they both contain the mitochondrial protein TOMM20, the synaptosome extract also contains the synaptic vesicle protein SV2 (Fig. 2c). However, extra-synaptic mitochondria are also present in the isolated synaptosome preparations. Excluding the substrates malate and succinate, as well as ADP, from the reaction assay minimizes the contribution of free extra-synaptic mitochondria to the OCR values measured in synaptosome isolates. OCR measurements in isolated synaptosomes were therefore carried out using pyruvate and glucose as the substrates in XF Base Media containing 1.8 mM calcium^36^. Since the synaptosome fraction also contains extrasynaptic mitochondria, membrane fragments, and related cellular material, SV2 was used as a proxy measure of the synaptic vesicle content within each preparation. Isolated synaptosome OCR results were normalized using SV2 immunoreactivity measured in each sample by capillary western^37,38^. Representative OCR curves obtained using equal amounts of synaptosomes from trained and untrained animals are shown in Fig. 2d. Similar to the data obtained with synaptic layer tissue punches, the basal (*t* = 4.693, *p* < 0.001), ATP-linked (*t* = 2.614, *p* < 0.001*)*, and maximal (*t* = 4.972, *p* < 0.001) respiration rates, were significantly increased in hippocampal synaptosomes isolated from the hippocampi of trained animals (Fig. 2e).

Previous studies have indicated that the frequency and/or the intensity of activity at a given synapse is associated with the number and activity of presynaptic mitochondria, and that increased activity enhances the localization of presynaptic mitochondria^7,39–42^. As the training-related increase in maximal respiration we observed could have arisen from an increase in the synaptic localization of mitochondria, synaptosomes were spotted on coverslips and double-label immunohistochemistry carried out to examine colocalization of the mitochondrial protein TOMM20 and the synaptic vesicle protein SV2 (Fig. 2f). Quantification of the number of synaptosomes containing a mitochondria did not reveal any significant difference in colocalization between the trained versus untrained rats (*t* = 173.00, *p* = 0.369; Fig. 2g).

### Drp1 function is required for learning-associated increase in OCR and memory formation

**Figure 3:**
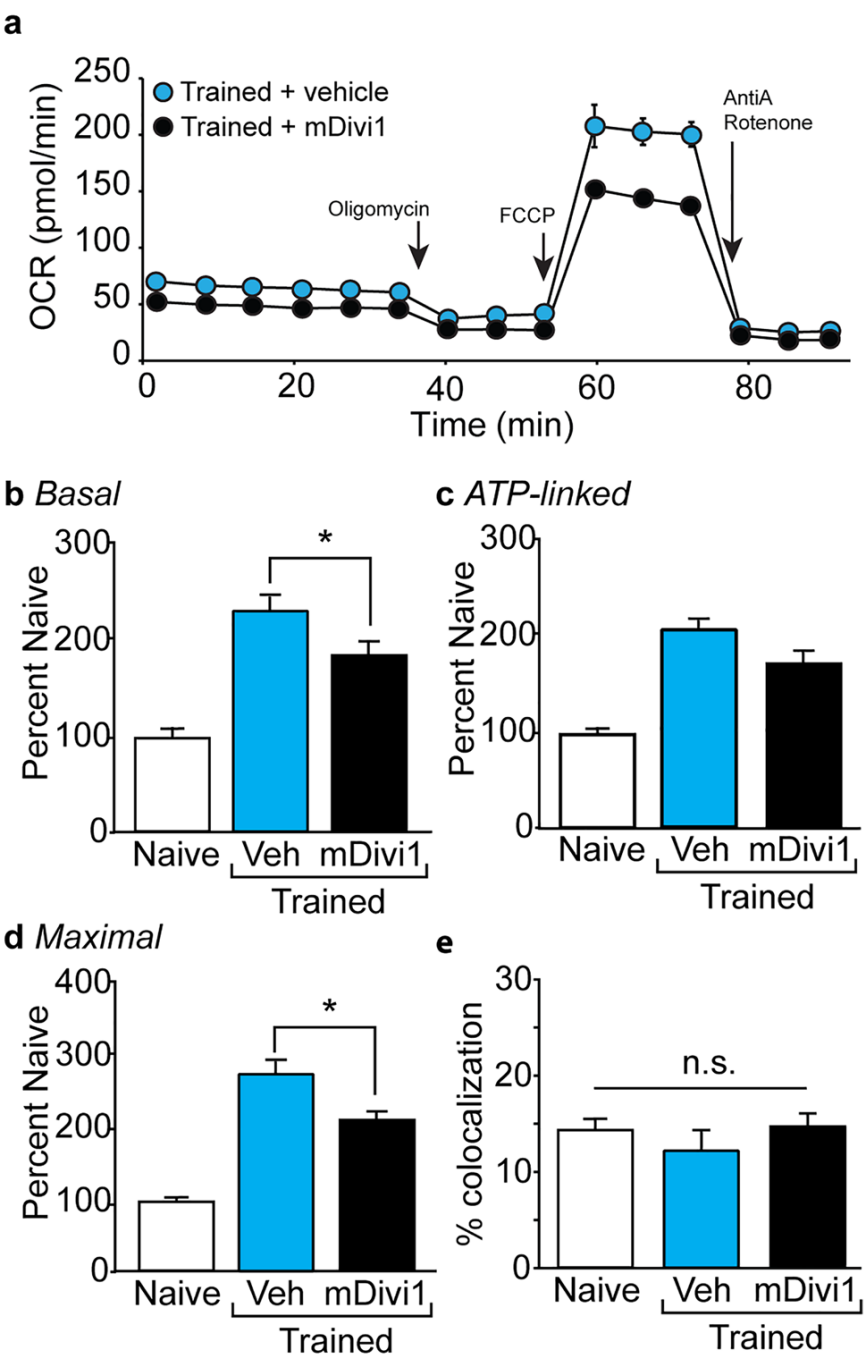
mDivi1 reduces training-related increases in synaptic respiration. (**a**) Representative OCR curves generated from hippocampal synaptosomes isolated from rats infused with either 3 µg/side of mDivi1 or vehicle (icv) prior to training in the contextual fear task. Summary data showing (**b**) basal, (**c**) ATP-linked, and (**d**) maximal respiration in hippocampal synaptosomes isolated from naïve, fear-trained rats pretreated with vehicle, and fear-trained rats pretreated with mDivi1 (n = 6/group). (**e**) Summary data showing the percentage of synaptosomes containing mitochondria in preparations isolated from naïve (n = 5), fear-trained rats pretreated with vehicle (n = 4), and fear-trained rats pretreated with mDivi1 (n = 6). Data are presented as mean ± SEM. *p < 0.05.

Mitochondrial fission is a process through which mitochondria can increase their number, reduce the size of mitochondria for transport, and increase energy production. Drp1 has been shown to be required for fission^23,27–29^, and Drp1 activity can be blocked by the cell membrane permeable inhibitor mDivi1^43,44^. We therefore questioned if mDivi1 can block the increased respiration seen in synaptosomes isolated from hippocampi of animals that have undergone fear training. Groups of animals (n = 6/group) received bilateral intracerebroventricular (icv) administration of 3 µg/side of mDivi1 (or an equal volume of 30% DMSO). Three hours following infusion, animals were trained in the context fear task and hippocampal synaptosomes isolated one hour later. The representative OCR curves presented in Fig. 3a show that rats that received pre-training infusions of mDivi1 had reduced basal and maximal respiration compared to rats that received vehicle. Comparisons across groups revealed significant training-related changes in basal (*F* = 49.7, *p* < 0.0001; Fig. 3b), ATP-linked (*F* = 23.3, *p* < 0.0001; Fig. 3c) and maximal (*F* = 72.5, *p* < 0.0001; Fig. 3d) respiration as compared to untrained controls. Post-hoc analysis showed that mDivi1-infused trained animals had significantly reduced basal (*p* = 0.001) and maximal (*p* = 0.003) respiration compared to vehicle-infused trained rats. These changes were not associated with a corresponding reduction in the number of synaptosomes containing a mitochondrion as no differences were detected across groups (Fig. 3e; *F* = 1.804, *p* = 0.207).

**Figure 4:**
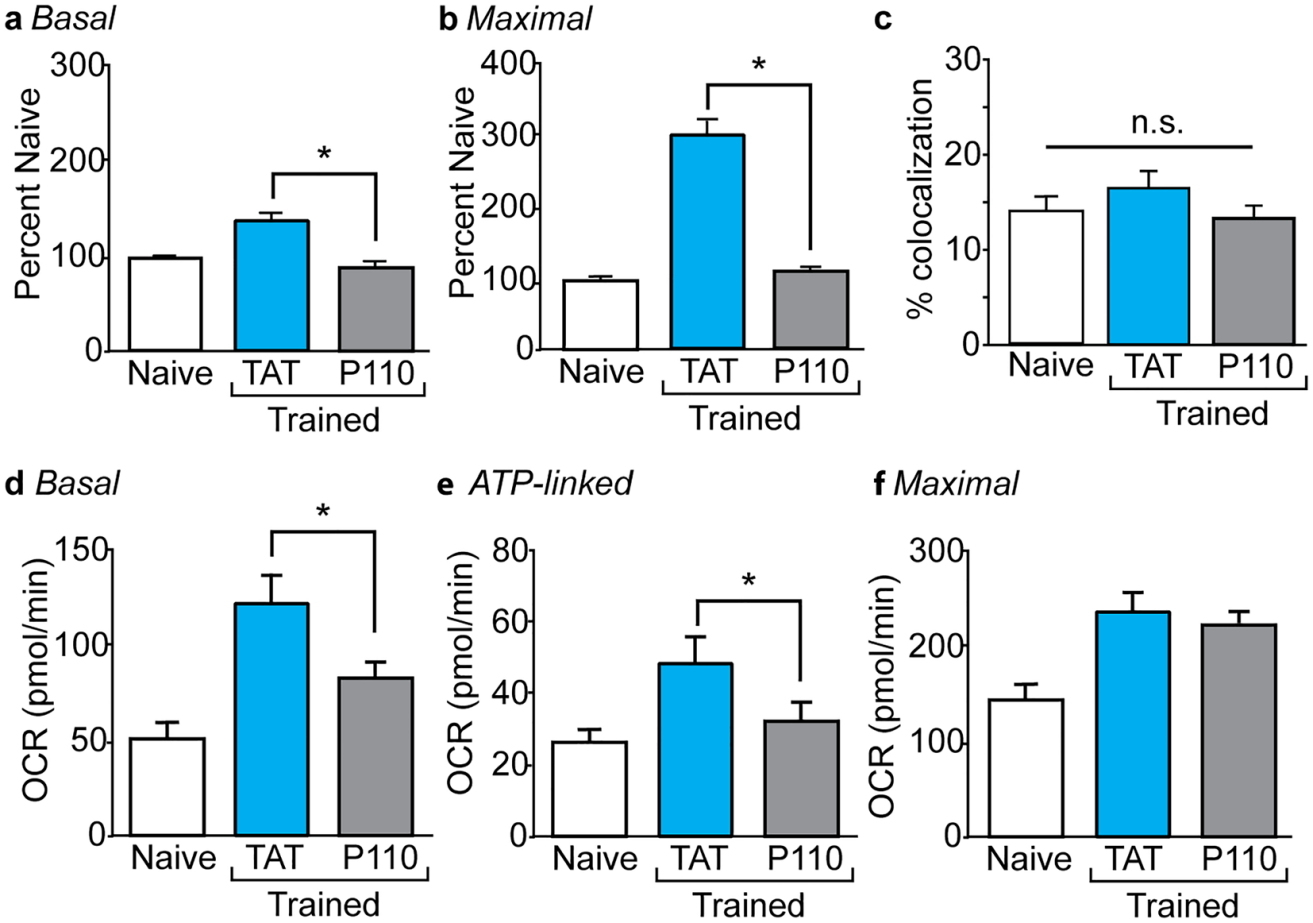
TAT-P110 reduces training-related increases in synaptic respiration. Summary data showing (**a**) basal and (**b**) maximal respiration in hippocampal synaptosomes isolated from naïve, fear-trained rats pretreated with TAT alone, and fear-trained rats pretreated with TAT-P110 (n = 6/group). (**c**) Summary data showing the percentage of synaptosomes containing mitochondria in preparations isolated from naïve (n = 5), fear-trained rats pretreated with TAT alone (n = 8), and fear-trained rats pretreated with TAT-P110 (n = 12). Summary data showing (**d**) basal, (**e**) ATP-linked, and (**f**) maximal respiration in SR/SLM tissue punches isolated from naïve, fear-trained rats pretreated with TAT peptide, and fear-trained rats pretreated with TAT-P110 peptide (n = 6/group). Data are presented as mean ± SEM. *p < 0.05.

To corroborate these findings, we next utilized a well-characterized cell-permeable peptide (TAT-P110) that inhibits Drp1 enzyme activity and blocks Drp1 interaction with fission protein 1 (Fis1)^45–48^. Groups of animals (n = 6/group) received bilateral icv infusions of 90 µg/side TAT-P110, or an equimolar amount of a TAT peptide control, dissolved in saline, and then given three hours for recovery before they were trained in the context fear task. Hippocampal synaptosomes were isolated one hour after training and used for OCR measurements. Comparisons across groups again revealed significant training-related increases in basal (*F* = 14.0, *p* < 0.0001; Fig. 4a) and maximal (*F* = 287, *p* < 0.0001; Fig. 4b) respiration. Post-hoc analysis showed that TAT-P110-infused trained animals had significantly reduced basal (*p* < 0.001) and maximal (*p* < 0.001) respiration compared to rats that received the control TAT peptide infusion prior to fear training. No significant change in the number of synaptosomes containing a mitochondrion was observed in response to TAT-P110 infusion (Fig. 4c; *F* = 1.494, *p* = 0.246).

To examine if similar effects could be observed in hippocampal tissue punches, independent sets of rats (n = 6/group) were icv infused with 90 µg/side TAT-P110 peptide or an equimolar amount of a TAT control peptide, and then given three hours for recovery before they were trained in the context fear task. Tissue punches were then taken from the SR/SLM beneath the CA1 subfield 1 h after context fear training. Figure 4d–f shows that basal (*F* = 12.48, *p* < 0.001; Fig. 4d), ATP-linked (*F* = 3.690, *p* = 0.031; Fig. 4e), and maximal (*F* = 11.65, *p* < 0.001; Fig. 4f) respiration were significantly different between the experimental groups. Post-hoc comparisons found that trained rats infused with TAT-P110 had significantly lower basal (*p* = 0.019) and ATP-linked (*p* = 0.047) respiration compared to trained rats that received the control TAT peptide infusion prior to training. In contrast to what we observed with isolated synaptosomes, TAT-P110 infusion did not significantly reduce maximal respiration in SR/SLM tissue punches (*p* = 0.565).

**Figure 5:**
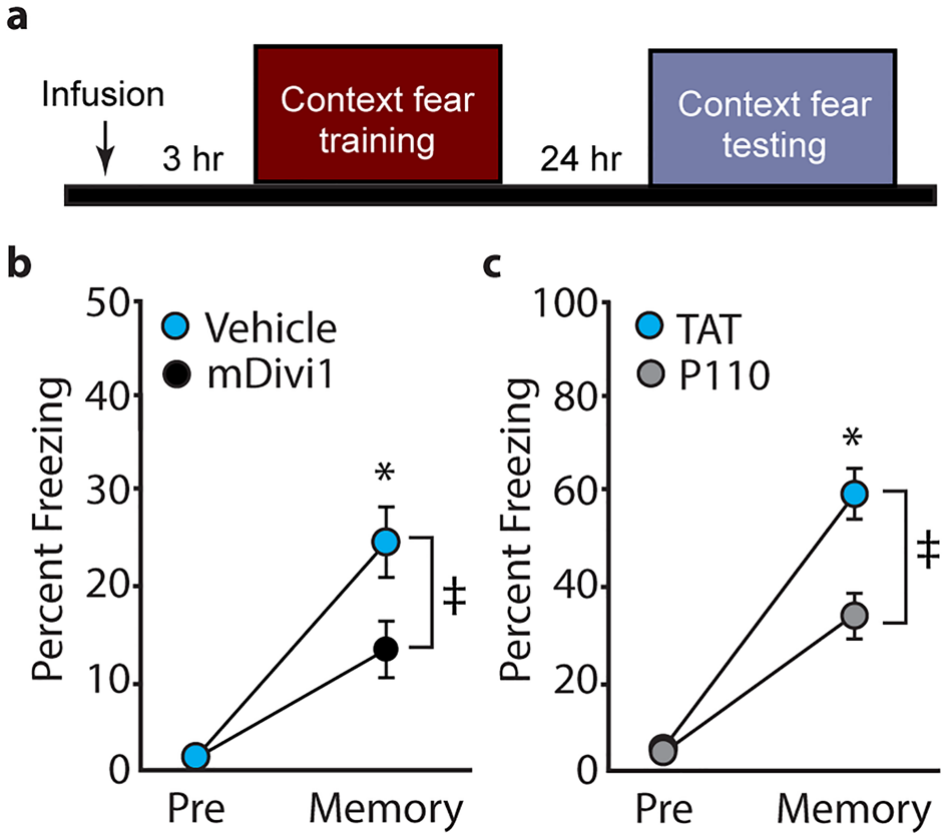
Drp1 Inhibitors impair context fear memory. (**a**) Schematic diagram showing the timeline of drug administration, fear memory training and testing. (**b**) Freezing behaviors manually recorded in rats treated with either mDivi or vehicle prior to training in a contextual fear task. While both groups showed minimal fear to the training chamber prior to the footshock (pre), freezing behaviors were enhanced 24 h post-training, indicating intact fear memory (memory). Rats pretreated with mDivi1 displayed less freezing behavior in the long-term memory test, indicating poor contextual memory compared to vehicle controls. (**c**) Freezing behaviors automatically recorded in rats treated with either TAT-P110 or TAT prior to training in a contextual fear task. Data are presented as mean ± SEM. ^‡^Significant interaction by repeated measures two-way ANOVA.

As OCR measurements indicated that fear training increased mitochondrial respiration in the SR/SLM synaptic layers of the hippocampus, and that pretreatment with mDivi1 or TAT-P110 peptide attenuated this increase, we questioned if pre-training mDivi1 administration would also alter memory formation. Prior to training, animals received bilateral intraventricular infusions of either mDivi1 (final concentration of 20 µM assuming equal distribution throughout the hippocampus) or an equal volume of vehicle. Three hours later, animals were trained in the context fear task. Long-term memory was tested 24 h later by measuring the percent time the animal remained in a freezing posture (absence of movement except that required for respiration) when placed back into the training chamber (Fig. 5a). The summary data (n = 12/group) presented in Fig. 5b shows that while both groups showed enhanced fear (compared to pre-shock levels) when placed back into the training chamber, rats that received pre-training mDivi1 had impaired contextual fear memory as indicated by reduced freezing behavior compared to rats that received the vehicle control (interaction of % freezing and session: *F* = 5.489, *p* = 0.029). Similar effects on long-term contextual memory were observed in rats (n = 8/group) pretreated with 90 µg TAT-P110 compared to those receiving an equimolar amount of TAT control peptide (Fig. 5c; *F* = 7.752, *p* = 0.015). Please note that in Fig. 5b, freezing behavior was hand scored by a blinded observer whereas in Fig. 5c, freezing behavior was calculated based on movements recorded using the activity monitoring module of *Ethovision XT* (Noldus, Leesburg, VA). The memory impairing effect of Drp1 inhibitor administration was specific to the training chamber as the percent freezing in a novel, otherwise non-threatening chamber, was not influenced by the treatments (data not shown).

## Discussion

In the present study, we measured mitochondrial respiration in hippocampal tissue punches and hippocampal synaptosome isolates prepared from fear-trained and control animals. In addition, we evaluated the role of mitochondrial fission protein Drp1 in learning-associated changes of mitochondrial respiration and in memory formation. Our analysis revealed three key findings: (1) learning of a hippocampus-dependent task increased tissue respiration in hippocampal synaptic layers, but not in CA1 cell bodies, (2) training-associated increased respiration was observed in hippocampal synaptosome preparations, and (3) inhibitors of the mitochondrial fission protein Drp1 attenuated the learning-associated increase in respiration, and impaired long-term fear memory. Together, our results indicate that behavioral training enhanced mitochondrial respiration at presynaptic sites in a Drp1-dependent manner that was independent of fission-related recruitment of mitochondria to presynaptic terminals.

Multiple ATP-consuming processes are involved in neuroplasticity and synaptic function. For example, Na^+^/K^+^ ATPase function is critical for maintaining the ionic gradients across the neuronal plasma membranes that are required for the generation and propagation of action potentials, and is the largest consumer of ATP in a neuron^3,49^. Endocytic processes necessary for regenerating synaptic vesicles, for buffering calcium, and for the loading and release of synaptic vesicles, also require mitochondrial function^8,22,25,26,50^. In addition, a large amount of ATP can be found within synaptic vesicles where it acts as a fast excitatory neurotransmitter by binding to post-synaptic purinergic P2X receptors^51^. Finally, synaptogenesis, which is a characteristic feature of long-term memory, is also an intensive energy-consuming process^16,17,52^. Although these and other studies have demonstrated a role for mitochondria in plasticity-related processes, presumably necessitating an acute increase in mitochondrial ATP production, whether synaptic mitochondrial respiration is increased in response to behavioral training in animals had not been demonstrated. Using tissue punches taken from the SR/SLM, we observed that contextual fear training increased synaptic, but not somatic, respiration. However, immunostaining of hippocampal synaptosome preparations showed that the number of synaptosomes (SV2^+^) that co-localized with mitochondria (indicated by TOMM20^+^ immunostaining) did not reveal any significant difference between trained and untrained animals. While the immunohistochemical detection approach we used cannot rule out the possibility that fission may have occurred after training within synaptosomes already containing a mitochondrion, our findings suggest that the increase in synaptic respiration we observed may result from other mechanisms, such as reorganizing respiratory complex proteins into supercomplexes, and/or altered post-translational modifications of mitochondrial proteins^53–56^. Future studies will be required to differentiate between these possibilities, and to identify the signaling pathways critical for the respiratory changes we observed.

As respiration measurements of synaptic layer biopsy tissue could not resolve if the observed training-associated increase in respiration resulted from increased respiration in the post-synaptic (CA1 dendritic) or presynaptic compartment, we measured OCR in isolated synaptosomes. As the primary constituents of synaptosome preparations are presynaptic terminals and free mitochondria, we used pyruvate and glucose in the presence of 1.8 mM calcium to minimize the contribution of free mitochondria to OCR^36^. Using these assay conditions, OCR measurements (normalized using SV2 levels as detected by western) revealed that training-associated increased respiration could be recapitulated in synaptosomes isolated from trained animals. Taken together, our results indicate that fear training does not increase energy production cell-wide, but rather enhanced presynaptic mitochondrial energy production, likely restricted to the presynapatic terminals of neurons engaged in memory formation.

As indicated above, it has been reported that there is increased mitochondrial fission in dendrites following stimulation, an event that can be blocked by manipulating Drp1^19,27^. We did not observe an increase in Drp1 phosphorylation in hippocampal extracts prepared from sham versus fear-trained animals (Supplemental Fig. S2). However, when rats were icv infused with either mDivi1 or TAT-P110 peptide infused prior to training, both synaptic respiration and long-term memory were impaired when compared to control animals that received icv vehicle injections (30% DMSO or TAT peptide alone, respectively). Although the directionality of change was consistent between the synaptosome, mDivi, and TAT-P110 results, there were small differences in the magnitude of OCR change observed between experiments. Differences in handling during housing, the drug/vehicle combinations, and/or sample preparations between the groups of animals used in each experiment may account for some of the variability in effect magnitude observed across experiments. Furthermore, synaptosome and tissue respiration experiments required different normalization approaches (SV2 content for synaptosome OCR, punch thickness/diameter for tissue OCR), which also may have contributed to the magnitude differences observed between experiments.

Our results indicate that while Drp1 function is required for enhanced mitochondrial respiration after training, Drp1 inhibitors did not alter the number of presynapatic terminals containing a mitochondrion. Previous experiments examining the function of hippocampal CA1 neurons lacking Drp1 showed that energy stress conditions impaired ATP generation at nerve terminals but not cell bodies, even though axonal mitochondria numbers were not changed^22^. This deficit in nerve terminal ATP generation impacted synaptic vesicle recycling and the releasable pool of synaptic vesicles. Although presynaptic terminals have a large reservoir of ATP, even transient ATP synthesis disruptions can alter synaptic function^18^. It remains to be determined if altered synaptic vesicle release in the presynaptic terminals may have contributed to the memory-impairments we observed in animals administered Drp1 inhibitors.

Synaptic vesicle release is triggered by an increase in presynaptic calcium resulting from the arrival of an action potential^57,58^. This presynaptic calcium needs to be sequestered to allow for subsequent action potentials and further neurotransmitter release. Calcium sequestration is most likely carried out by the endoplasmic reticulum and mitochondria located near (or in) the presynaptic terminal. A number of studies have reported that calcium uptake by mitochondria can alter cristae structure^59–61^, which may have implications for ATP synthesis^8,53,62,63^. For example, a recent study has shown that MICU1, via interaction with the MICOS complex, plays an important in regulating mitochondrial cristae dynamics^64^. Respiratory complexes are localized within a crista in order to facilitate proton accumulation and to maximize the electromotive force needed to drive ATP synthesis. It remains to be determined if learning is associated with alterations in the cristae of presynaptic mitochondria, and if Drp1 plays a role in these changes^65,66^.

## Methods

### Animals

All experimental procedures were conducted in accordance with the Guide for the Care and Use of Laboratory Animals of the National Institutes of Health and were approved by the Institutional Animal Care and Use Committee (IACUC) using procedures consistent with those outlined in the ARRIVE guidelines. Male Sprague–Dawley rats (275–300 g) were purchased from Envigo (Houston, Texas). Rats were group housed on a 12-h light/dark cycle, with ad libitum access to food and water. All experiments were performed during the light cycle.

### Intracerebroventricular (icv) infusion

Anesthesia was induced with 5% isoflurane carried by 1:1 N_2_O/O_2_ mixture in an induction chamber and then maintained with a 2.5% isoflurane carried by 1:1 O_2_/air mixture via a face mask. Animals were mounted on the stereotaxic frame secured by ear bars and an incisor bar. The head was held in a horizontal plane with respect to the interaural line and middle sagittal suture. A midline incision was made and the skull exposed. For infusion, two small burr holes were prepared over the lateral ventricles (0.80 mm posterior to bregma, ± 1.5 mm lateral from midline). A 32 gauge infusion needle was lowered via a stereotaxic arm to a depth of 3.6 mm from the skull surface. The icv infusion rate was kept at 0.5 μl/min for the entire 10 min infusion period. After infusion, the needle was held in position for 1 min before being withdrawn from the ventricle. Core body temperature was maintained at 37 °C using a heating pad coupled to a rectal thermometer. After the completion of the infusion, the incision was closed with wound clips and animals were allowed to completely recover from the anesthesia in a warm chamber before being placed back in their home cages.

### Context fear conditioning

Context fear conditioning was carried out essentially as previously described^67^. For tissue punch and synaptosome mitochondrial respiration experiments, a naïve control group was used for baseline comparisons. These naïve rats were maintained in their home cages until euthanized for tissue preparations and not exposed to the training chamber (in the absence of foot shock) to avoid the potential confound of novel environment exploratory learning. Rats that underwent training were placed in the training chamber and given three consecutive training sessions, each consisting of a 2 min exploratory period followed by a 2 s, 0.70 mA foot shock, and remained in the training chamber for the duration of training protocol. Thirty seconds after training, the rats were returned to their home cage, then 1 h after completion of training quickly euthanized. One hemisphere was sliced and used for tissue punch respiration, while the hippocampus was quickly dissected from the remaining hemisphere and used for synaptosome isolation and respiration measurements (see below).

For experiments assessing behavior, control animals received vehicle injection (30% DMSO for mDivi experiments; saline containing the TAT peptide without the P110 cargo for TAT-P110 experiments), and experimental animals received drug injection, 3 h prior to training in a one-foot shock version of the context fear paradigm^67^. Both the vehicle-injected and drug-injected animal groups received one-trial context fear training. This training paradigm generates a moderate amount of freezing behavior during testing, allowing for either increased or decreased fear behavior to be observed. Thirty seconds after the shock, rats were removed from the training chamber and returned to their home cages. Twenty-four hours later, memory was tested by placing the animals back into the training chamber and recording their freezing behavior either manually by a blinded observer, or by using an automated tracking device (Noldus, Wageningen, Netherlands), over a 3 min testing period. To examine the specificity of the learned fear, rats were also tested in a novel environment that shared certain features with the training chamber (e.g. size, smell), but differed in others (e.g. wall panel color, flooring).

### Preparation of brain slices

Rats were humanely euthanized, brains were rapidly removed (within 30 s of decapitation) and immersed in ice-cold (4–5 °C) artificial cerebrospinal fluid (aCSF; 120 mM NaCl, 3.5 mM KCl, 1.3 mM CaCl_2_, 1 mM MgCl_2_, 0.4 mM KH_2_PO_4_, 5 mM HEPES, and 10 mM D-glucose; pH 7.4) that had been oxygenated for 1 h using 95% O_2_:5% CO_2_. Coronal sections (200 μm) were prepared using a modified McIlwain tissue chopper (Ted Pella. Inc.; Redding, CA) with a chilled stage and blade, then transferred to a holding chamber containing continuously oxygenated aCSF at room temperature (~ 23 °C).

### Tissue punches and respiration measurements

Brain sections were individually transferred to a biopsy chamber containing fresh oxygenated aCSF. A stainless steel WellTech Rapid-Core biopsy punch needle (500 µm diameter; World Precision Instruments; Sarasota, FL) was used to excise the tissue punches. Tissue punches were taken from each anatomical location using four consecutive coronal sections (*i.e.*, a total of 4 punches for each anatomical structure) using the same biopsy punch needle. Punches were ejected directly into an XFe96 Cell Culture Microplate (101085-004; Agilent Technologies, Santa Rosa, CA) based on a pre-determined plate layout. Each well contained 180 µL room temperature assay media (aCSF supplemented with 0.6 mM pyruvate and 4 mg/mL lyophilized BSA). After loading all biopsy samples, each well was visually inspected to ensure that the punch was submerged and centered at the bottom. The XFe96 Cell Culture Microplate was then incubated at 37 °C for approximately 30 min. During this incubation period, 10 × concentration of assay drugs (prepared in aCSF) were loaded into their respective injection ports of a hydrated (overnight in distilled water, exchanged for XF Calibrant solution 3 h prior to assay initiation) Seahorse XFe96 Extracellular Flux Assay sensor cartridge. The sensor cartridge containing the study drugs was then inserted into the analyzer for calibration. Once the analyzer was calibrated, the calibration plate was replaced by the microplate containing the tissue punches and the assay protocol initiated. Assay drugs were prepared at 10 × working concentrations in oxygenated aCSF (pH 7.4) and delivered sequentially to achieve final concentrations of: port A: Oligomycin (25 µg/mL); port B: FCCP + pyruvate (75 µM and 7.5 mM, respectively); and port C: Antimycin A + rotenone (10 µM and 5 µM, respectively). The dose of each of these reagents was based on optimization experiments to achieve optimum drug effect^30^. The duration of sampling time (for calculating the oxygen consumption rate or OCR) for each condition was determined to allow the effect of each drug to reach a steady state. Wells with low basal activity (< 20 pmol/min OCR) and/or failing to respond to FCCP/pyruvate were excluded from analysis.

Although we found that citrate synthase activity was directly proportional to the number of tissue punches, the activity in a single tissue punch was near the limit of detection (see supplemental Fig. S1). Furthermore, since the reaction buffer contained BSA, measuring total protein content would be inappropriate. Therefore, tissue respiration results were normalized following the procedures recommended by the Seahorse XF analyzer manufacturer. The tissue sections were cut to a uniform thickness (200 µm), and all punches within an experiment were prepared using the same biopsy needle (500 µm diameter). We have previously shown that there is a linear relationship between tissue punch diameter and OCR for brain biopsies ≤ 1.0 mm in diameter^30^. In addition, care was taken to ensure that the anatomical locations of the punches were consistent across sections and animals. OCR data from at least 4 punches/anatomical structure/animal was collected.

### Synaptosome isolation and respiration measurements

Synaptosomes were isolated using Percoll density gradient centrifugation^68^. Briefly, after euthanasia, the brain was rapidly removed and hemisected. Hippocampal tissue from one hemisphere was quickly dissected and homogenized in ice-cold GM base buffer (250 mM sucrose, 5 mM Tris pH 7.4, 0.1 mM EDTA, 1 mM sodium fluoride, 1 mM sodium molybdate, 100 nM okadaic acid, 1 mM PMSF and 10 µg/ml leupeptin) using a Dounce homogenizer. Tissue was homogenized using 8–10 strokes with the loose pestle (72–120 µm clearance), followed by 8–10 strokes using the tight pestle (20–56 µm clearance). The homogenate was centrifuged at 1000×*g* for 10 min at 4 °C. The supernatant was then layered onto a discontinuous Percoll gradient (3%, 10%, 15%, and 23% Percoll) and centrifuged for 10 min (30,700×*g* at 4 °C). The synaptosome fraction at the 15—23% interface was recovered and transferred to individual centrifuge tubes, washed with GM buffer, pelletized by centrifugation (16,700×*g* at 4 °C) for 10 min, and used for respiration assays.

Extrasynaptic mitochondria, in addition to fragmented membranes and other cellular material, are present in the synaptosome fraction^68^. Sample to sample normalization using citrate synthase activity (which would detect extra-synaptic mitochondria), or total protein content (which would detect non-synaptic cellular material), are not desirable. SV2, which is selectively expressed on synaptic vesicles, was therefore used as a proxy for the synaptosome content within each preparation. The level of the synaptic vesicle protein SV2 in each synaptosome preparation was measured by capillary western (as performed previously by us^37,38^), and used to normalize the OCR data.

For synaptosome respiration measurements, pyruvate (10 mM) and glucose (15 mM) were added to Agilent’s XF Base Media (pH 7.4, no phenol red), and the resulting solution used to make 10 × drug stocks. The stock solutions were loaded into the drug ports of a hydrated sensor cartridge in the following order: (A) oligomycin (2.5 µg/mL final), (B) FCCP (4 µM final), and (C) antimycin A (4 µM final) + rotenone (2 µM final). Prior to plating the synaptosomes, the XFe96 cell culture microplate was coated overnight at 37 °C with 20 µL/well PEI (0.003% solution). The plate was aspirated and put on ice 30 min prior to assay. Isolated synaptosomes (1 µg/well) were plated on the coated Seahorse cell culture microplate in 20 µL of MAS + substrate + 0.2% w/v fatty-acid free BSA and centrifuged at 1000×*g* for 60 min at 4 °C. The assay medium (XF Base Media + substrates + 0.2% BSA) was then added to the wells to bring the final volume to 180 µL prior to the plate being incubated at 37 °C for 30 min and transferred to the analyzer for analysis. The respiration assay protocol consisted of a 2 min mix, 2 min wait, and 3 min measure cycle.

### Electron microscopy

For Cryo-electron microscopy, freshly isolated synaptosomes were applied to glow-discharged carbon-coated copper grids then vitrified^69^. Images of isolated synaptosomes were acquired by an experimenter blind to the treatment group using a G2 Polara cryo-electron microscope operated at 300 kV in low dose mode. Images were collected in photon-counting mode on a Gatan K2 Summit direct electron detector.

### Immunohistochemistry and mitochondria localization

Synaptosomes were diluted (1:1250 to 1:5000) in isolation buffer and centrifuged 1500×*g* for 30 min at 4 °C onto polyethylenimine (PEI)-treated coverslips. The supernatant was gently aspirated, and the samples fixed for 5 min at 4 °C in PBS containing 4% paraformaldehyde. Coverslips were permeabilized for 5 min with PBS containing 0.5% Triton X-100, blocked for 30 min with PBS containing 2.5% BSA, 2.5% goat serum, and 0.1% Tween-2, and stained for 1 h with blocking buffer containing 1 µg/ml primary antibodies (all steps done at RT). Synaptosomes were identified by the presence of the presynaptic vesicle protein SV2 (mouse IgG1 anti-SV2; Developmental Studies Hybridoma Bank), mitochondria were detected using the mitochondrial-specific marker TOMM20 (rabbit anti-TOMM20; Abcam #ab186734), and the primary antibodies were detected using appropriate host-specific fluorescent secondary antibodies. Images from the stained coverslips (6 × 6 grid) were captured using a Zeiss LSM 510 confocal microscope equipped with a 40X oil objective. The total number of synaptosomes (SV2^+^) and those containing mitochondria (colocalized SV2^+^ and TOMM20^+^) were counted using the image analysis module in Zen Blue 2.6.

### Citrate synthase activity

Groups of rats (n = 5/group) were either trained in the conditioned fear task or remained in their home cages. One hour after training the animals were quickly euthanized, brains removed under ice-cold aCSF, sectioned, and biopsy punches collected from the SR/SLM and cortex as described above. Five SR/SLM punches per animal were collected, pooled, snap frozen on dry ice, and stored at – 80 °C until needed. Cortical biopsy punches (between 1 and 30 biopsies per sample) were also collected and used to construct a standard curve. Citrate synthase enzyme activity was measured using the MitoCheck assay kit (Cayman Chemical, cat.# 701040) following the manufacturer’s protocol. Briefly, biopsy samples were sonicated in 150 µl assay buffer, and 30 µl lysate used per assay well (assayed in duplicate). Reactions were initiated by the addition of an oxaloacetate solution, and the absorbance at 412 nm was recorded every 20 s for 25 min. The reaction rate for each well was calculated from the linear portion of the assay curve, and values averaged for each animal.

### Capillary westerns

Target protein levels in tissue homogenates, synaptosome preparations, or tissue biopsy samples were measured using an automated capillary immunoassay system (Wes system; Protein Simple, San Jose, CA). For biopsy punches, a common reference pool was generated using equal volumes of protein lysate from each sample, and a serial dilution series was used to generate standard curves for the target proteins. Samples were diluted into 1X Wes sample buffer, and the relative abundance of the target proteins (Drp1, cell signaling 8570; phosphoDrp1, cell signaling 4867; Tomm20, abcam ab186734; OxPhos mitochondrial antibody cocktail for SDHB/complex I; UQCRC2/complex II, MTCO1/complex III; ATP5a/complex V, ThermoFisher #45-8099) were quantified. Immunoreactivity was detected with a luminol-peroxide solution, and a series of timed exposures were collected. The images were analyzed using Compass for SW (version 3.1.7; Protein Simple).

### Statistical analysis

All data were subjected to a Shapiro–Wilk normality test to ensure a normal distribution. Respiration, mitochondria localization, and enzyme assay results were evaluated using either one-way ANOVAs or t-tests depending on the number of groups being compared. For data that did not have a normal distribution, a one-way ANOVA on Ranks (or Mann–Whitney rank sum test for two sample comparisons) was used. The Holm-Sidak method for multiple comparisons post-hoc test was used to determine the data points with significant differences. For comparing freezing behaviors in the context fear task, a two-way repeated measures ANOVA was used to examine differences across groups across scoring sessions. Data were considered significant at *p* < 0.05 and presented as mean ± standard error of the mean (SEM).

### Supplementary Information

Supplementary Figures.

## Data Availability

All data generated or analyzed during this study are included in this published article (and its Supplementary Information files).
