# Supplementary material for: Enhanced presynaptic mitochondrial energy production is required for memory formation
Source: Sci Rep. 2023 Sep 2;13:14431. doi: 10.1038/s41598-023-40877-0 (PMC10475119; doi:10.1038/s41598-023-40877-0)
Supplement: Supplementary file 1 — Supplementary Figures. [file 41598_2023_40877_MOESM1_ESM.pdf]

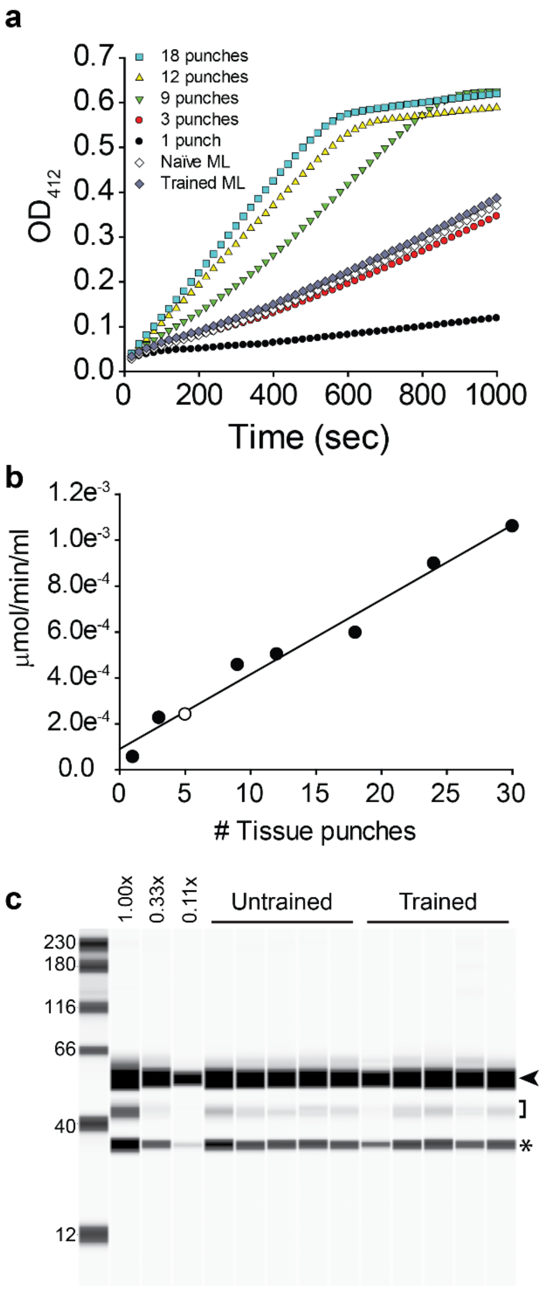

Supplemental Figure 1. Citrate synthase activity and expression of mitochondrial complex proteins are not altered by fear-training. Our tissue respiration data indicated that fear training increases mitochondrial respiration in punches taken from the CA1 synaptic layer (SR/SLM). It is possible that an increase in mitochondrial mass could have given rise to the increase we observed. To assess this possibility, we examined citrate synthase activity and the expression levels of representative mitochondrial core proteins. Groups of animals (n=5/group) were trained in a three trial version of the context fear conditioning task, or were maintained in their home cages. One hour after training, animals were euthanized, brains quickly dissected under ice-cold artificial CSF, and 200 µm sections prepared for tissue biopsy isolation. SR/SLM punches were pooled (5 punches/animal) and snap frozen until assayed. Varying numbers of cortical biopsy punches were pooled to

generate a reference standard curve. To determine citrate synthase activity, the pooled tissue punches were directly lysed in 150 µl reaction buffer (MitoCheck citrate synthase activity assay kit; Cayman Chemical, cat# 701040) and enzyme activity determined according to the manufacturer's protocol. **a)** Change in absorbance over time is shown for the standard curve, and for representative untrained and fear-trained SR/SLM samples. **b)** The slope (determined from the linear portion of each curve) was used to calculate the enzymatic activity for each sample and plotted versus the number of pooled biopsy punches. Citrate synthase activity was directly proportional to tissue input. Filled circles: citrate synthase activity as a function of number of pooled cortical punches. Open circle: enzyme activity for pooled SR/SLM tissue punches (5 punches/pool). **c)** Lysates of SR/SLM tissue punches (diluted 1:2) from untrained and fear-trained rats (5 punches/pool) were used for western analysis to quantify the expression levels of mitochondrial respiratory complex core proteins. Equal volumes of lysate were separated by capillary western and probed with a cocktail of mouse monoclonal antibodies (ThermoFisher catalogue #45-8099) directed against complex II (SDHB, *), complex III and complex IV (UQCRC2 and MTCO1, bracket; these proteins do not adequately separate by WES), and complex V (ATP5a, arrowhead). A standard curve of serially diluted lysate was simultaneously prepared and probed to determine the linearity of the immunoreactivities. Full-length capillaries are shown.

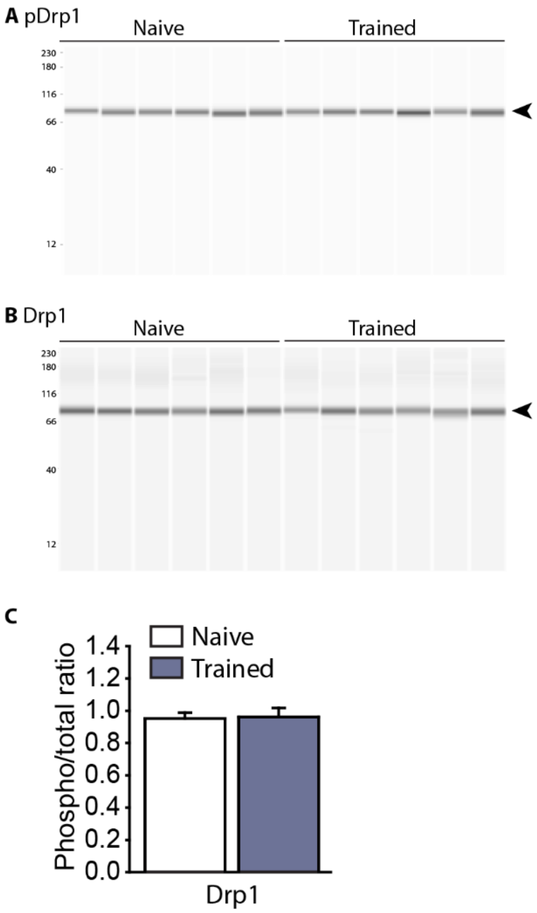

Supplemental Figure 2. Fear training does not cause a global increase in hippocampal Drp1 phosphorylation. Drp1 Ser616 phosphorylation has been shown to increase mitochondrial fission [68,69]. Groups of animals (n=6/group) were trained in a three trial version of the context fear conditioning task, or were maintained in their home cage (Naïve). One hour after training, rats were euthanized and hippocampi quickly dissected under ice-cold artificial CSF. Hippocampal extracts were prepared in the presence of protease and phosphatase inhibitors. Equal amounts of protein were separated on a *Wes* capillary western system (Bio-Techne, Minneapolis, MN) and probed for **a**) phosphorylated Drp1 (Ser616), and **b**) total Drp1 immunoreactivity. Full-length capillaries are shown. **c**) Calculation of the ratio of the phosphorylated Drp1/total Drp1 immunoreactivities did not reveal a significant influence of training on hippocampal Drp1 phosphorylation ($t = -0.444$, $p = 0.666$).

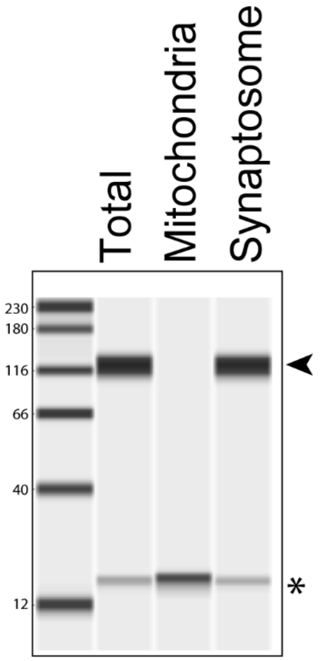

Supplemental Figure 3. Expanded full-length capillary westerns from Figure 2c. Capillary westerns showing synaptic (SV2, arrowhead) and mitochondrial (TOMM20, asterisk) immunoreactivity in total homogenate, isolated mitochondria, and isolated synaptosomes. The mitochondrial marker TOMM20 was found in all samples, while the vesicle marker SV2 was detected in only the total and isolated synaptosome samples. A longer exposure of the image shown in Figure 2c is depicted here to clearly show the capillary tube edges. The box indicates the edges of the full-length, uncropped image captured using the Wes system (Protein Simple, San Jose, CA).

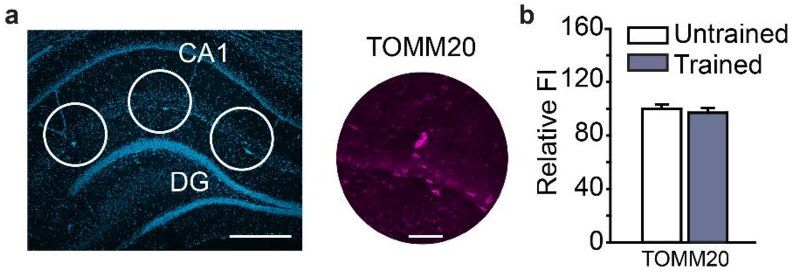

Supplemental Figure 4. Groups of animals (n=5/group) were trained in a three trial version of the context fear conditioning task, or were maintained in their home cage (untrained). One hour after training, rats were euthanized and tissues quickly sectioned into 200 µm thick sections followed by fixation in 4% paraformaldehyde for 30 minutes. Following cryoprotection, tissue sections (30 µm in thickness) were prepared on a cryostat. Mitochondrial mass was assessed by examining the immunoreactivity of TOMM20. The fluorescent intensity values in three 500 µm regions (centered on the SR/SLM) were average for each tissue section. Three tissue sections/animal were independently stained and quantified. **a)** Representative image of a bisbenzamide-stained tissue section (scale bar = 500 µm) showing the relative positions of the three regions used for quantifying TOMM20 immunoreactivity. A representative field of view of TOMM20 immunoreactivity is also shown (scale bar = 100 µm). **b)** Summary data (n=5 animals/group) showing no change in TOMM20 immunoreactivity was observed in the SR/SLM between untrained and fear–trained animals.
